# Vascular endothelial growth factor (VEGF) polymorphism rs3025039 and atherosclerosis among older with hypertension

**DOI:** 10.1038/s41598-022-09486-1

**Published:** 2022-04-01

**Authors:** Yuji Shimizu, Kazuhiko Arima, Yuko Noguchi, Hirotomo Yamanashi, Shin-Ya Kawashiri, Kenichi Nobusue, Fumiaki Nonaka, Kiyoshi Aoyagi, Yasuhiro Nagata, Takahiro Maeda

**Affiliations:** 1 Department of General Medicine, Nagasaki University Graduate School of Biomedical Sciences, Nagasaki, Japan; 2 Department of Cardiovascular Disease Prevention, Osaka Center for Cancer and Cardiovascular Diseases Prevention, Osaka, Japan; 3 Department of Public Health, Nagasaki University Graduate School of Biomedical Sciences, Nagasaki, Japan; 4 Leading Medical Research Core Unit, Nagasaki University Graduate School of Biomedical Sciences, Nagasaki, Japan; 5 Department of Community Medicine, Nagasaki University Graduate School of Biomedical Sciences, Nagasaki, Japan; 6 Department of Islands and Community Medicine, Nagasaki University Graduate School of Biomedical Sciences, Nagasaki, Japan

**Keywords:** Biomarkers, Predictive markers, Angiogenesis, Risk factors

## Abstract

Angiogenesis inhibition therapy causes hypertension by increasing peripheral vascular resistance. Vasa vasorum angiogenesis plays a crucial role in the development of atherosclerosis. Since vascular endothelial growth factor (VEGF), which contributes to the progress of angiogenesis, is reported to be inversely associated with the minor allele of polymorphism rs3025039, the minor allele of rs3025039 could be inversely associated with atherosclerosis among individuals with hypertension. A cross-sectional study of 1793 older Japanese adults aged 60–89 years with hypertension who participated in general health check-ups was conducted. Atherosclerosis was defined as carotid intima-media thickness (CIMT) ≥ 1.1 mm. The minor allele of polymorphism rs3025020 was positively associated with VEGF. Therefore, in addition to known cardiovascular risk factors, rs3025020 genotype acted as a confounding factor in the present study. Independent of known confounding factors, the minor allele of rs3025039 was inversely associated with atherosclerosis among older Japanese adults with hypertension. The fully adjusted odds ratio (OR) and 95% confidence interval (CI) for atherosclerosis with the minor allele of rs3025039 was 0.78 (0.64, 0.96). The angiogenesis-related polymorphism rs3025039 was associated with the development of atherosclerosis among older Japanese individuals. This study indicates that the development of atherosclerosis among older individuals might partly indicate a capacity for angiogenesis.

## Introduction

Vascular endothelial growth factor (VEGF) plays an important role in angiogenesis^1^. A previous study reported that the serum concentration of VEGF is positively associated with the minor allele of the VEGF polymorphism rs3025020 and inversely associated with the VEGF polymorphism rs3025039^2^. Since VEGF inhibition therapy induces hypertension^3^ while the minor allele of VEGF rs3025020 is inversely associated with hypertension^4^, lower VEGF activity might induce hypertension by disrupting the microcirculation through less angiogenesis. On the other hand, vasa vasorum angiogenesis is an important process that leads to structural atherosclerosis^5^.

Those studies suggest contradictions about the role of angiogenesis in hypertension and atherosclerosis. Even though hypertension is reported to be positively associated with atherosclerosis (CIMT ≥ 1.1 mm)^6^, blocking angiogenesis causes hypertension^1,3^ and angiogenesis plays an important role in the development of atherosclerosis^5^.

Aging is a process that increases the level of oxidative stress^7^. Oxidative stress is a known cause of hypertension^8^ and atherosclerosis^7^.

Therefore, clarifying the association between the VEGF polymorphism rs3025039, which might have undesired effects in the progression of angiogenesis, and atherosclerosis among older individuals with hypertension could be an efficient way to clarify the role of angiogenesis-related genetic characteristics in the development of atherosclerosis.

To evaluate the association between the VEGF polymorphism rs3025039 and atherosclerosis in the context of hypertension, we conducted a cross-sectional study of 1793 Japanese patients with hypertension who participated in general health check-ups in 2017–2019.

## Material and methods

### Study population

The methods related to the present risk surveys, including genetic data (polymorphism), have been described elsewhere^4,6^.

Considering the shortage of staff to conduct health checkups in the present survey, the entire population of the city could not be surveyed in the span of 1 year. Therefore, we conducted the survey in different parts of Goto City over a period of 3 years to ensure that all areas of the city were included. Details about the present study have been described elsewhere^9^.

The study population was comprised of 2828 individuals (1066 men and 1762 women) aged 60–89 years from Goto City in western Japan who had previously attended an annual health check-up conducted by the local government under the direction of the Ministry of Health, Labor and Welfare of Japan during 2017–2019.

Participants without data on rs3025039 genotype (n = 134) or rs3025020 genotype (n = 6) were excluded. We also excluded participants without hypertension (n = 895). The remaining 1793 elderly Japanese individuals with hypertension (692 men and 1101 women with a mean age of 74.3 ± standard deviation (SD) 7.1 years) were enrolled in the study.

Written consent forms were made available to ensure that the participants understood the objective of the study. Informed consent was obtained from all participants. All procedures performed in studies involving human participants were in accordance with the ethical standards of the institutional research committee and with the 1964 Helsinki declaration and its later amendments or comparable ethical standards. This study was approved by the Ethics Committee for Human Use of Nagasaki University (project registration number 14051404-11).

### Data collection and laboratory measurements

Trained interviewers obtained the medical history and habitual status of each participant. Body weight and height were measured using an automatic body composition analyzer (BF-220; Tanita, Tokyo, Japan), from which body mass index (BMI; kg/m^2^) was calculated.

Blood pressure (systolic and diastolic) was measured in the sitting position using a blood pressure measuring device (HEM-907; Omron, Kyoto, Japan) after at least 5 min of rest. High blood pressure was defined as systolic blood pressure (SBP) ≥ 140 mmHg, diastolic blood pressure (DBP) ≥ 90 mmHg, or both. If a participant had high blood pressure, we measured blood pressure a second time. The lower blood pressure values were used. Hypertension was defined as SBP ≥ 140 mmHg, DBP ≥ 90 mmHg, or use of antihypertensive medication.

Fasting blood samples were collected. Concentrations of triglycerides, HDL cholesterol (HDLc), and hemoglobin A1c (HbA1c) were measured using standard laboratory procedures. All measurements were performed by SRL, Inc. (Tokyo, Japan).

Genomic DNA was extracted from 2 mL of whole peripheral blood using Gene Prep Star NA-480 (Kurabo Industries Ltd., Osaka, Japan). Genotyping of the single nucleotide polymorphisms (SNPs) rs3025039 and rs3025020 was conducted using TaqMan assays and a LightCycler 480 thermal cycling platform (Roche Diagnostics, Basel, Switzerland).

### Measurement of carotid intima-media thickness (CIMT)

Experienced vascular technicians measured CIMT using a LOGIQ Book XP device with a 10-MHz transducer (GE Healthcare, Milwaukee, WI, USA). The maximum CIMT values for the left and right common carotid arteries were calculated using digital edge-detection software (Intimascope; Media Cross, Tokyo, Japan) according to a previously described protocol^10^.

The maximum values of right and left CIMT, which did not include plaque measurements, were then calculated. The maximum CIMT value was used for analysis. Since a previous study reported CIMT < 1.1 mm was normal, we defined atherosclerosis as CIMT ≥ 1.1 mm^11–13^.

### Statistical analysis

The characteristics of the study participants in relation to rs3025039 genotype were expressed as means ± SD for continuous variables except for triglycerides. Since triglycerides had skewed distributions, triglycerides were expressed as medians [interquartile range] and logarithmic transformation was used for analysis. Gender distribution, daily drinking status, smoking status, anti-hypertensive medication status, and genotype of rs3025020 (C/T and T/T) were expressed as n (%). A trend test was performed using a regression model.

Logistic regression was used to calculate odds ratios (ORs) and 95% confidence intervals (CIs) to determine associations between rs3025039 genotype and atherosclerosis. Three different models were used to adjust for confounding factors. Model 1 adjusted only for sex and age. Model 2 further adjusted for rs3025020 genotype because a previous study reported that the serum concentration of VEGF is positively associated with the minor allele of the VEGF polymorphism rs3025020 and inversely associated with the VEGF polymorphism rs3025039)^2^. In Model 3, we included the variables in Model 2 plus other potential confounding factors, such as BMI (kg/m^2^), drinking status (none, often, daily), smoking status (no, yes), triglycerides (mg/dL), HDLc (mg/dL), and HbA1c (%). In order to validate the study population in the present study, goodness of fit was evaluated using the Hosmer–Lemeshow test.

All statistical analyses were performed using the SAS system for Windows (version 9.4; SAS Inc., Cary, NC). A *p* value < 0.05 was considered statistically significant.

## Results

### Characteristics of study population

Table 1 shows the characteristics of the study participants by rs3025039 genotype. CT-heterozygote and TT-homozygote status for rs3025020 were significantly inversely associated with the minor allele of rs3025039, respectively.

**Table 1:** Characteristics of study participants by rs3025039 genotype.

	rs3025039 genotype			*p* value
	C/C	C/T	T/T	
No. of participants	1181	513	99	
Men, %	37.8	39.4	43.4	0.500
Age, years	74.5 ± 7.1	73.8 ± 7.0	73.5 ± 7.6	0.062
Daily drinker, %	15.4	19.3	17.2	0.141
Smoker, %	6.8	7.2	9.1	0.675
BMI, kg/m^2^	23.6 ± 3.5	23.5 ± 3.3	23.6 ± 3.4	0.774
SBP, mmHg	145 ± 17	143 ± 18	145 ± 18	0.129
DBP, mmHg	79 ± 12	78 ± 13	81 ± 12	0.141
Antihypertensive medication, %	69.0	71.5	59.6	0.060
Triglycerides, mg/dL	92[67, 129]*^1^	90[74, 131]*^1^	86[65, 131]*^1^	0.328*^2^
HDLc, mg/dL	61 ± 15	61 ± 14	60 ± 15	0.790
HbA1c, %	5.8 ± 0.5	5.8 ± 0.6	5.7 ± 0.5	0.283
rs3025020 (C/T), %	42.9	35.5	8.1	< 0.001
rs3025020 (T/T), %	12.7	0.2	0.0%	< 0.001

Values are means ± standard deviation unless otherwise indicated.

*BMI* body mass index, *SBP* systolic blood pressure, *DBP* diastolic blood pressure, *HDLc* HDL cholesterol, *HbA1c* hemoglobin A1c.

*^1^Values are median [the first quartile, the third quartile].

*^2^Logarithmic transformation was used for evaluating *p*.

### Association between rs3025039 genotype and atherosclerosis

Table 2 shows the association between rs3025039 genotype and atherosclerosis. The minor allele of rs3025039 was inversely associated with atherosclerosis among older Japanese patients with hypertension. The adjusted OR and 95% CI for atherosclerosis with the minor allele of rs3025039 was 0.77 (0.63, 0.95) in the model that adjusted for age and sex (Model 1), 0.78 (0.64, 0.96) in the model that further adjusted for rs3025020 status (Model 2), and 0.78 (0.64, 0.96) in the model that further adjusted for known cardiovascular risk factors (Model 3). Goodness of fit was validated for the present study population (*p* ≥ 0.05) (not shown in table); *p* = 0.916 for Model 1, *p* = 0.669 for Model 2, and *p* = 0.302 for Model 3.

**Table 2:** Odds ratios (ORs) and 95% confidence intervals (CIs) for atherosclerosis based on rs3025039 genotype.

	rs3025039 genotype			P for trend	Minor allele (T)
	C/C	C/T	T/T		
Number of participants	1181	513	99		
Number of cases (%)	295 (25.0)	114 (22.2)	12 (12.1)		
Model 1	Reference	0.89 (0.69, 1.14)	0.41 (0.22, 0.77)	0.012	0.77 (0.63, 0.95)
Model 2	Reference	0.90 (0.69, 1.16)	0.42 (0.22, 0.79)	0.007	0.78 (0.64, 0.96)
Model 3	Reference	0.90 (0.70, 1.17)	0.41 (0.22, 0.78)	0.021	0.78 (0.64, 0.96)

Model 1: adjusted only for sex and age. Model 2: adjusted for variables in Model 1 plus rs3025020 genotype. Model 3: adjusted for variables in Model 2 plus BMI, drinking status (none, often, daily), smoking status (no, yes), triglycerides, HDLc, and HbA1c.

*HDLc* HDL cholesterol, *HbA1c* hemoglobin A1c, *Cases* atherosclerosis.

### Sensitivity analysis

For sensitivity analysis, we performed sex-specific analysis for the association between rs3025039 genotype and atherosclerosis. We found essentially the same associations; the age-adjusted OR (95% CI) for atherosclerosis with the minor allele of rs3025039 was 0.81 (0.61, 1.08) for men (n = 692) and 0.75 (0.57, 0.99) for women (n = 1101). Goodness of fit was validated for the present study population (*p* ≥ 0.05) (not shown in table); *p* = 0.266 for men and *p* = 0.364 for women, respectively.

Since the present study deals with genetic factors, age indicates the duration of exposure to the risk factor of interest (rs3025039). For a sensitivity analysis that takes duration exposure into consideration, analyses stratified by age group were performed. The associations were essentially the same. With rs3025039 (C/C) as the referent group, sex- and age-adjusted ORs and 95% CIs of atherosclerosis with rs3025039 (C/T) and rs3025039 (T/T) were 0.83 (0.48, 1.44) and 0.16 (0.02, 1.21) in the age 60–69 group (n = 569), 1.09 (0.77, 1.56) and 0.48 (0.19, 1.17) in the age 70–79 group (n = 783), and 0.61 (0.37, 0.99) and 0.50 (0.18, 1.41) in the age 80–89 group (n = 441), respectively. Goodness of fit was validated for the present study population (*p* ≥ 0.05); *p* = 0.467 for the age 60–69 group, *p* = 0.956 for the age 70–79 group, and *p* = 0.587 for the age 80–89 group, respectively.

## Discussion

The major finding of the present study involving older Japanese patients with hypertension is that the minor allele of the VEGF-related polymorphism rs3025039 is inversely associated with atherosclerosis. Since the associations were essentially the same in the sex-specific analysis, sex might not have affected the present results.

And because the associations were essentially the same in the age group stratified analysis, duration of exposure to the risk factor of interest (rs3025039) also might not have affected the present results.

Previously, the minor allele of the VEGF polymorphism rs3025020 was reported to be positively associated with serum VEGF levels^2^ and inversely associated with hypertension, possibly as an indicator of higher angiogenesis activity^4^. The minor allele of the VEGF polymorphism rs3025039 was reported to be inversely associated with serum VEGF levels^2^.

In the present study, we found further evidence that, among older individuals with hypertension, the minor allele of the VEGF polymorphism rs3025039 is inversely associated with atherosclerosis. A biological reaction programmed to counteract oxidative stress might be underlying this inverse association.

Hypoxia increases oxidative stress^14,15^. Aging is a process that increases levels of peripheral hypoxia^16^ and oxidative stress^17^. Increased levels of oxidative stress are critical for living bodies^18^. The body has two major ways to overcome the influence of hypoxia and oxidative stress. The first involves a mechanism to compensate for blood flow. Hypertension and angiogenesis contribute to this compensatory blood flow. Hypertension increases the effectiveness of the existing vascular system and angiogenesis creates new vascular architecture. The second method is to increase the productivity of anti-oxidative agents. Increasing hemoglobin production might be a mechanism that reduces oxidative stress by generating chemicals that counteract oxidative stress. Details about these mechanisms regarding a biological reaction programmed to counteract oxidative stress have been described elsewhere^9^.

Reduction of oxidative stress is a purpose that is common to hypertension and angiogenesis. Therefore, with more oxidative stress, hypertension and active angiogenesis could both be observed. However, if the process of angiogenesis is sufficient to reduce oxidative stress, hypertension is no longer necessary and vice vasa^6^. Analysis among older individuals with hypertension could enhance the understanding of how angiogenesis deficiency affected on development of atherosclerosis. The process of vasa vasorum angiogenesis is necessary for the development of structural atherosclerosis^5^.

The minor allele of rs3025039 is reported to be inversely associated with serum VEGF levels^2^. VEGF plays an important role in the development of angiogenesis^1^. Therefore, the minor allele of rs3025039 could be inversely associated with the development of atherosclerosis because of lower levels of vasa vasorum angiogenesis.

This study with CIMT evaluation showed that genetic characteristics associated with reducing the activity of angiogenesis also might block the development of atherosclerosis. Since inhibition of angiogenesis induces hypertension^3^, a process that prevents the development of atherosclerosis could induce hypertension, even though hypertension is generally regarded to be positively associated with atherosclerosis (CIMT ≥ 1.1 mm)^6^. Therefore, the present findings have some crucial clinical implications, namely that no development of atherosclerosis as evaluated by CIMT could also act as an indicator of no development of angiogenesis and the induction of hypertension.

Impairment of the microcirculation might contribute to lower muscle strength^19^. Platelets play an important role in angiogenesis^20^ and endothelial repair^21^. Platelet count could indicate vascular repair activity^22^. Since atherosclerosis is a process of aggressive endothelial repair, the presence of atherosclerosis in participants with high platelet count might indicate more angiogenesis and endothelial repair activity. Our previous study with hypertensive Japanese individuals aged 60–89 years showed a positive association between muscle strength (handgrip strength) and atherosclerosis (CIMT ≥ 1.1 mm) only in subjects with high platelet counts (platelet ≥ median)^12^. Furthermore, among hypertensive Japanese men aged 60–89 years, muscle strength (tongue pressure) is inversely associated with atherosclerosis (CIMT ≥ 1.1 mm) in those with low platelet levels, but not in those with high levels^13^. Those studies indicate that the development of atherosclerosis (CIMT ≥ 1.1 mm), which is associated with more angiogenesis and endothelial repair activity, could contribute to the maintenance of muscle strength among hypertensive Japanese aged 60–89 years, which is compatible with our present study that showed genetic characteristics associated with a disadvantage in maintaining the microcirculation (angiogenesis) might lead to a lower chance of developing atherosclerosis in hypertensive older aged 60–89 years.

Furthermore, hematopoietic stem cells, also known as CD34-positive cells, contribute to both higher CIMT^23–25^ and the development of angiogenesis^26^. VEGF promotes the migration and differentiation of CD34-positive cells^27^.

However, among older men aged 60–69 years, hypertension reduces the number of CD34-positive cells via consumption during activated endothelial repair^24,25^. This finding also indicates that the development of atherosclerosis as evaluated by CIMT could indicate sufficient residual capacity for angiogenesis among older individuals with hypertension.

The minor allele carrier of rs3025039 has a disadvantage in the development of angiogenesis^1,2^. Serum concentration of VEGF which plays important role in angiogenesis^1^ is inversely associated with the miner allele of rs3025039 and positively associated with rs3025020^2^. Since angiogenesis contributes to maintain microcirculation, further investigation with information of microcirculation status is necessary to perform linkage disequilibrium analysis for rs3025039 and rs3025020.

Potential limitations of the present study warrant consideration. First, we have no data on plasma VEGF values because of blood sample volumes were limited. Further investigation with serum VEGF data is necessary. Because there are many unknown genetic factors that influence angiogenesis, further investigation is necessary. Even though we thought that oxidative stress might play an important role in the present results, we have no data to allow for oxidative stress evaluation. Further study with reactive oxygen species (ROS) is necessary.

Independent from known potential confounding variables, the minor allele of rs3025039 is inversely associated with atherosclerosis among older with hypertension. This finding suggests an efficient way to clarify the mechanism of vascular maintenance among older individuals.
